# Intraoral and transcutaneous cervical ultrasound in the differential diagnosis of peritonsillar cellulitis and abscesses

**DOI:** 10.1016/S1808-8694(15)30972-1

**Published:** 2015-10-19

**Authors:** Bernardo Cunha Araujo Filho, Flavio A. Sakae, Luiz Ubirajara Sennes, Rui Imamura, Marcus R. de Menezes

**Affiliations:** a PhD Student – Otorhinolaryngology – University Hospital – Medical School of the University of São Paulo; Otorhinolaryngologist (former resident - HCFMUSP), Specialist from the ABORL-CCF; b PhD Student - Otorhinolaryngology - Otorhinolaryngologist; c Associate Professor – Department of Otorhinolaryngology – Medical School of the University of São Paulo. Head of the Department of Oral-phyryngology – HCFMUSP; d Assistant Physician – PhD – Department of Otorhinolaryngology – University Hospital – Medical School of the University of São Paulo. Head of the Otorhinolaryngology Emergency Room - HCFMUSP; e MD. PhD, Assistant physician – Department of Radiology – Medical School of the University of São Paulo. Radiologist. Study carried out at the Otorhinolaryngology Department and Radiology Department of the University Hospital – Medical School of the University of São Paulo

**Keywords:** peritonsillar abscess, peritonsillar cellulitis, ultrasound

## Abstract

**Aims:**

The objective of the present study was to determine the specificity, sensitivity and accuracy of intraoral and transcutaneous ultrasound (US) in the diagnosis of peritonsillar cellulitis and abscess.

**Study Design:**

Clinical-Prospective.

**Materials and Metods:**

Thirty nine patients were seen at the otorhinolaryngology emergency department of the University Hospital, of the School of Medicine, University of São Paulo, with a clinical diagnosis of peritonsillar cellulitis or abscess. After initial evaluation, all patients were submitted to intraoral and transcutaneous US.

**Results:**

Intraoral US was performed on 35 cases and its sensitivity was of 95.2%, the specificity was of 78.5% and the accuracy was of 86.9%. Transcutaneous US was feasible in all 39 patients and diagnosed peritonsillar abscess in 53.8%. There were 5 false-negatives and 1 false-positive result, sensitivity was 80%, specificity was 92.8% and accuracy was 84.5%.

**Conclusions:**

Intraoral US was quite sensitive in the diagnosis of peritonsillar abscesses when performed by an experienced radiologist. Specificity was higher for transcutaneous US compared to intraoral US. However, when transcutaneous US was performed in patients with trismus, it was able to diagnose all peritonsillar abscesses, since they were large collections which are common in patients with trismus. These exams showed similar accuracy.

## INTRODUCTION

The peritonsillar space is located between the palatine tonsil fibrous capsule (medially) and the fascia of the superior constrictor muscle (laterally), being the most common site of abscess formation in the head and neck^1^. It is typically more common in adolescents and young adults resulting from propagation of tonsillar infections, which lead to cellulitis or peritonsillar abscesss^2^. If treated incorrectly, the abscess may cause severe consequences for patients such as aspiration and pneumonia, as well as deep cervical infection with serious consequences, such as mediastinitis, sepsis and even morte^1^, ^3^, ^4^, ^5^. Clinically, peritonsillar abscesses and cellulitis have a similar presentation that is almost impossible to differentiate based on the clinical history and the physical examination^3^, ^6^, ^7^. Differentiation between these two entities, which are part of the same illness, is essential for successful treatment. Peritonsillar abscesses (PAs) may be treated with needle aspiration, drainage of pus or tonsillectomy, while cellulitis (PC) is treated with antibiotics^3^, ^7^, ^8^. The differential diagnosis between cellulitis and peritonsillar abscesses is made by needle aspiration and careful aspiration of the peritonsillar space^8^, ^9^. Frequently repeated needle aspiration is needed to locate the possible abscess. This procedure is painful and risky, there is the possibility of injuring blood vessels such as the internal carotid artery, and it may be difficult in children and patients with significant trismo^1^, ^8^, ^10^, ^11^. An abscess may not be diagnosed in some patients, which results in inadequate treatment^7^. Ultra-sound (US) has been used in the diagnosis of abscesses since 1950; in past 15 years it has become much more frequently used in medical conditions.

In this context there have been attempts to develop and evaluate methods to make the correct differential diagnosis between PC and PA. There are references in literature on the use of intra-oral and transcutaneous US to differentiate PC or PA, since there is no correlation between the onset of the abscess and the duration of infecção^12^, however these studies had a limited number of patients and inexperienced radiologists to diagnose peritonsillar space infections. A comparison between intra-oral and transcutaneous US in the differential diagnosis of PC and PA has not yet been done.

The aim of this study was to establish the accuracy, specificity and sensitivity of intra-oral and transcutaneous US in the diagnosis of cellulitis and peritonsillar abscesses.

## CASES AND METHODS

**Figure 1:**
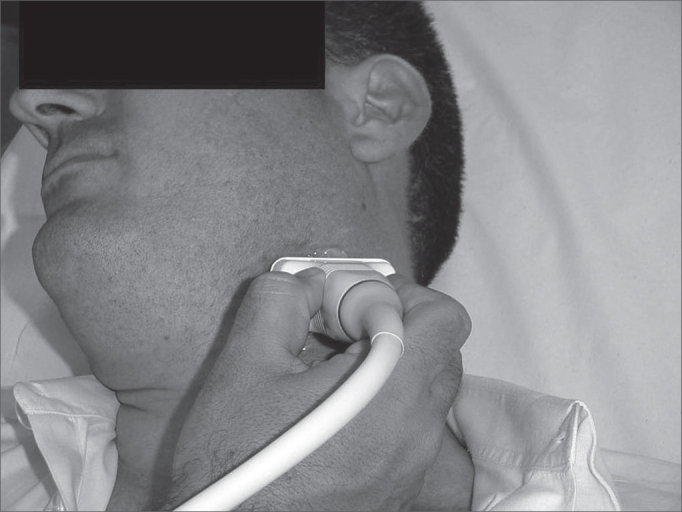
Transcutaneous US placed in the angle of the lower jaw with the patient in orthostatism and lateral rotation of the head.

**Figure 2:**
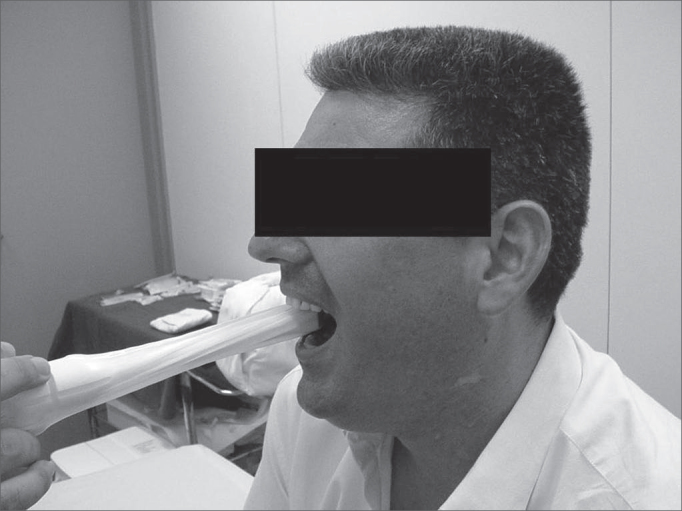
Intra-oral US done with the patient seated and with the mouth open, allowing contact between the intra-cavity transducer and the affected tonsil.

In this prospective study, thirty nine patients with a clinical diagnosis of cellulitis or peritonsillar abscess were attended at the emergency unit of the Clinical Hospital of the Sao Paulo University Medical School. The research protocol was approved by the Research Ethics Committee; participants read and signed a free and informed consent form. Twenty four were women and fifteen were men aged between 7 and 44 years. Following otorhinolaryngological evaluation, all patients underwent intra-oral and transcutaneous US by a radiologist familiar with the radiological diagnosis of this entity. The radiologist did not have access to the clinical hypothesis of cellulitis or abscess raised by the otorhinolaryngologist. We used a General Electric 500 ultrasound equipment (Milwakee, USA) with a 7,5Mhz central frequency linear transducer placed on the angle of the lower jaw of the patient in orthostatism and lateral rotation of the head (see Figure 1). Intra-oral US was done with a condom-covered 6,5Mhz intracavity transducer covered; the patient was seated with the mouth open and xylocaine 10% spray was applied to the oropharynx for anesthesia, so that the intracavity transducer could be placed over the affected tonsil (see Figure 2).

The patients were classified, according with ultrasonographic findings, as having cellulitis or a peritonsillar abscess, and the abscess volume was measured. The diagnosis was confirmed in all patients by needle aspiration with a jelco 14 needle on three points: the superior polar region, the middle polar region and the inferior polar region. If needle aspiration was positive, an incision and drainage were undertaken. If negative, the patients were diagnosed as having cellulitis and treated with antibiotics. Intra-oral and transcutaneous US were compared with needle aspiration results (see Table 1). We calculated the sensitivity, specificity, the negative predictive value and the positive predictive value of the two tests. We also made Receiver Operator Characteristic (ROC) curves of these tests and calculated the areas under the curves (accuracy). These areas were compared to check whether a test was more accurate that the other by using the Chi-Squared test.

**Table 1:** Results of abscess and cellulitis assessment in patients undergoing intra-oral US, transcutaneous US and needle aspiration.

Patient	Intra-oral US	Transcutaneous US	Needle aspiration
1	Abscess	Abscess	Abscess
2	Abscess	Abscess	Abscess
3	Not done (trismus)	Abscess	Abscess
4	Cellulitis	Cellulitis	Cellulitis
5	Abscess	Abscess	Cellulitis
6	Abscess	Abscess	Abscess
7	Abscess	Abscess	Abscess
8	Cellulitis	Cellulitis	Cellulitis
9	Cellulitis	Cellulitis	Cellulitis
10	Abscess	Cellulitis	Cellulitis
11	Cellulitis	Cellulitis	Cellulitis
12	Abscess	Abscess	Abscess
13	Abscess	Abscess	Abscess
14	Abscess	Abscess	Abscess
15	Abscess	Cellulitis	Abscess
16	Abscess	Abscess	Abscess
17	Not done (trismus)	Abscess	Abscess
18	Cellulitis	Cellulitis	Cellulitis
19	Cellulitis	Cellulitis	Cellulitis
20	Abscess	Cellulitis	Abscess
21	Not done (trismus)	Abscess	Abscess
22	Not done (trismus)	Abscess	Abscess
23	Cellulitis	Cellulitis	Cellulitis
24	Cellulitis	Cellulitis	Abscess
25	Abscess	Abscess	Abscess
26	Abscess	Abscess	Abscess
27	Abscess	Abscess	Abscess
28	Abscess	Cellulitis	Abscess
29	Abscess	Abscess	Abscess
30	Abscess	Abscess	Abscess
31	Cellulitis	Cellulitis	Cellulitis
32	Cellulitis	Cellulitis	Cellulitis
33	Cellulitis	Cellulitis	Cellulitis
34	Abscess	Cellulitis	Cellulitis
35	Abscess	Abscess	Abscess
36	Abscess	Cellulitis	Abscess
37	Abscess	Abscess	Abscess
38	Cellulitis	Cellulitis	Cellulitis
39	Abscess	Abscess	Abscess

## RESULTS

Intra-oral US could not be done in 4 patients out of 39 patients due to significant trismus. Intra-oral US found abscesses in 65.7% of cases and cellulitis in 34.3% of cases. Jelco needle aspiration was positive in 21 patients and negative in 14 patients. There were 3 false positive cases and 1 false negative case. Sensitivity was 95.2% and specificity was 78.5%. The positive predictive value was 87% and the negative predictive value was 91.7% (see Table 2). Transcutaneous US was done in all patients and diagnosed peritonsillar abscesses in 53.8% of cases. There were 5 false negative cases and 1 false positive case; sensitivity was 80% and specificity was 92.8% (see Table 3). The accuracy of transcutaneous US was 84.5% and the accuracy of intra-oral US was 86.9%, with no statistically significant difference (p = 0.72) (Chart I). There were bilateral abscesses in 1 case.

**Table 2:** Intra-oral US

	Positive	Negative	Total
Abscess	20(87%)	01(8,3%)	21(60%)
Cellulitis	03(13%)	11(91,7%)	14(40%)
Total	23 (100%)	12(100%)	35(100%)

**Table 3:** Transcutaneous US. Aspiration with jelco

	Positive	Negative	Total
Abscess	20(95,2%)	05(27,7%)	25 (64,1%)
Cellulitis	01(4,8%)	13(72,3%)	14 (35,9%)
Total	21(100%)	18(100%)	39 (100%)

**Chart 1:**
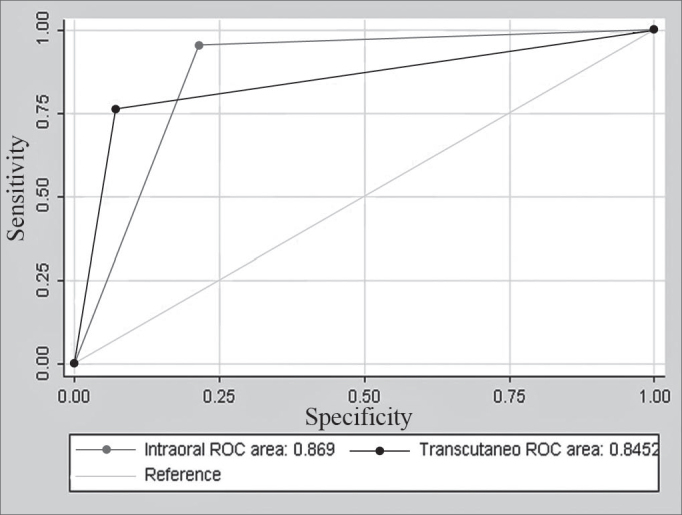
ROC (Receiver Operator Characteristic) curve for Intra-oral and Transcutaneous US.

## DISCUSSION

The origin of peritonsillar space inflammation is controversial; according to some authors, it initiates with infection of the Weber glands in the supra-tonsillar fossa1. Patients with peritonsillar abscess and cellulitis may present throat pain, fever, dysphagia, trismus, malaise and may progress unfavorably, developing aspiration pneumonia and extension of the infection into deep neck spaces, even leading to death in some cases^1^, ^6^, ^7^. Increases in the number of infections of the peritonsillar space have been observed in children due to the inadequate use of antibiotics^11^. Wide bore needle aspiration has been used to differentiate between PC and PA; it is a potentially dangerous, invasive and painful method^8^, ^10^. Haeggstrom et al.^8^ and Amhed et al.^9^ have suggested that needle aspiration is an accurate procedure for this diagnosis, in contrast to Scott et al.^7^, that reached 89% sensitivity compared with computerized tomography. Computerized tomography is effective to diagnose deep neck affections, however, it is expensive, subject to complications due to use of contrast and difficult to access in emergency units^1^, ^3^. We believe that systematic punctures of the superior, middle and inferior polar region of the tonsillar fossa may reduce false negatives; furthermore, in a clinical setting, this method has been effective to diagnose infection in the peritonsillar space. Miziara et al.^3^, in attempts to reduce the risks of unnecessary needle aspiration in patients, have evaluated the use of intra-oral US to diagnose abscesses and cellulitis, with a 92.3% sensitivity and a 62.5% specificity. Haeggstrom et al.^8^ reported an 85% success rate in the diagnosis of abscesses with intra-oral US. In our study, intra-oral US sensitivity was 95.2% and specificity was 78.5%, superior to the abovementioned studies. A larger sample size and an experienced radiologist were determining factors for the success of intra-oral US in differentiating between cellulitis and abscesses. Analyzing the 3 false positive cases and 1 false negative with intra-oral US, these occurred with small collections of liquid (< 1ml), similar to Ahmed^9^ e Strong's^6^ findings. Intense edema and inflammation may also lead to false positive results. Intra-oral US could not be done in 4 patients that had significant trismus. The sensitivity of transcutaneous US (80%) was lower compared to intra-oral US (92.5%) in the same patients, however it was more specific (92.8%) compared to intra-oral US (90%). Ahmed et al.^9^, in a study of 27 patients, reported a 90% sensitivity for transcutaneous US, better than our results, however inferior to the rate obtained with intra-oral US. Transcutaneous US had 5 false negative results, limiting its use to the diagnosis of peritonsillar abscesses. Small needle-aspirated collections of liquid (< 2.5ml) is also related to an increase in false negatives during transcutaneous US. We recommend that results of this exam be evaluated with care, as there was a tendency to underestimate the volume of collections of liquid. When intra-oral US could not be used due to trismus, transcutaneous US diagnosed abscesses in 100% of cases. The aspirated volume was increased in these patients (> 4ml), which is related to the more exuberant clinical picture and which was responsible for the increased sensitivity of transcutaneous US in these patients. In literature unilateral abscesses are observed in 93% of cases^2^, ^3^, similar to our percentage. Transcutaneous US was important in cases where intra-oral US was impossible. In our study transcutaneous US and intra-oral US together improved the diagnosis of PA and PC compared to Miziara et al.'s^3^ and Haeggstrom et al.'s^8^ rates. US also allowed the otorhinolaryngologist to guide needle puncture, avoiding multiple blind punctures in any one patient. This has also been credited to US by Sakagushi et al.^1^, Patel et al.^4^ and Blaivas et al.^5^ Although transcutaneous US was specific, some cases were not detected. When dealing with patients that have potential risks of serious complications, high false negative rates are unacceptable. A sensitive low false negative rate examination is needed for the diagnosis of peritonsillar space conditions. Transcutaneous US, a more specific exam, could be in cases where intra-oral US was impossible; used simultaneously, they would not increase diagnostic accuracy in peritonsillar infections.

## CONCLUSION

Intra-oral US was more sensitive but less specific than transcutaneous US in the diagnosis of peritonsillar abscesses, when undertaken by an experienced radiologist. It is an efficient and accurate method to differentiate the diagnosis of cellulitis and peritonsillar abscesses. Only transcutaneous US was possible in patients with trismus, being sufficiently sensitive in larger collections of liquid, usually seen in these patients. These examinations had statistically similar accuracy.
